# Apparent Negative Reflection with the Gradient Acoustic Metasurface by Integrating Supercell Periodicity into the Generalized Law of Reflection

**DOI:** 10.1038/srep38314

**Published:** 2016-12-05

**Authors:** Bingyi Liu, Wenyu Zhao, Yongyuan Jiang

**Affiliations:** 1 Department of Physics, Harbin Institute of Technology, Harbin 150001, China; 2 Key Lab of Micro-Optics and Photonic Technology of Heilongjiang Province, Harbin 150001, China

## Abstract

As the two dimensional version of the functional wavefront manipulation metamaterial, metasurface has become a research hot spot for engineering the wavefront at will with a subwavelength thickness. The wave scattered by the gradient metasurface, which is composed by the periodic supercells, is governed by the generalized Snell’s law. However, the critical angle that derived from the generalized Snell’s law circles the domain of the incident angles that allow the occurrence of the anomalous reflection and refraction, and no free space scattering waves could exist when the incident angle is beyond the critical angle. Here we theoretically demonstrate that apparent negative reflection can be realized by a gradient acoustic metasurface when the incident angle is beyond the critical angle. The underlying mechanism of the apparent negative reflection is understood as the higher order diffraction arising from the interaction between the local phase modulation and the non-local effects introduced by the supercell periodicity. The apparent negative reflection phenomena has been perfectly verified by the calculated scattered acoustic waves of the reflected gradient acoustic metasurface. This work may provide new freedom in designing functional acoustic signal modulation devices, such as acoustic isolator and acoustic illusion device.

Tailoring the wavefront into arbitrary desired shape with a metasurface, the two dimensional metamaterial with subwavelength inclusions, has attracted tremendous attention in recent years. Since metasurface itself can be regarded as the application of the Huygens’ principle, arbitrary wavefront manipulation can be realized by designing these artificial secondary sources according to the detailed information of the desired wavefront. The milestone work done by *Yu, N et al*.^1^ stimulates the intensive investigation about the electromagnetic metasurfaces and reveals the huge potentials underneath the metasurface of realizing highly integrated functional electromagnetic devices, such as the planar lens^2^,^3^, optical vortex generator^4^,^5^, holograms^6^,^7^ and ultrathin cloaking^8^,^9^. Some nontrivial physical phenomenon, such as the photonic spin Hall effect^10^,^11^ and the generalized laws of reflection and refraction^1^,^12^,^13^ to name a few, can be amply studied with the help of the gradient metasurfaces. Similar to their electromagnetic counterparts, acoustic metasurfaces have become attractive for they are able to engineer the phase profiles of the impinging waves by the artificial designed structures with subwavelength thickness instead of the space consuming solutions offered by the traditional diffractive acoustic devices. Numerous types of acoustic meta-atoms have been proposed to construct the functional acoustic metasurfaces, such as tapered labyrinthine structure^14^, coiling-up slit structure^15^,^16^, zigzag channel^17^, Helmholtz resonator array^18^, split sphere^19^ and membrane based structure^20^. Based on these acoustic meta-atoms, a great number of acoustic wavefront manipulation devices have been constructed and operated successfully, for example, the acoustic focusing lens^21^,^22^, acoustic vortex beam generator^23^, acoustic Airy beam generator^24^,^25^, acoustic carpet cloaking^26^,^27^ and so on.

Gradient metasurface is a periodic array of the supercell with a linearly varying phase modulation ranging from 0 to 2π, and the wave scattered by the gradient metasurface is governed by the generalized laws of reflection and refraction. According to the generalized laws of reflection and refraction, there exists a critical angle which defines the domain that the anomalous reflection or refraction can occur, and beyond the region defined by the critical angle the incident beam would be transformed into the surface bounded wave, and thus the metasurface works as the anti-scattering coating^28^,^29^. However, a recent study about the transmissive acoustic metasurface reports the observation of the apparent negative refraction when the incident angle is beyond the critical angle^30^ and a review paper also covers some detailed discussion about this interesting phenomena^31^. The apparent negative refraction investigated by *Xie Y. et al*. can be understood by revisiting the influence of the supercell periodicity as well as the local phase modulation on the acoustic gradient metasurface. Thereafter, a more comprehensive discussion about the negative refraction that occurs in optical region when the incident angle is beyond the critical angle has been demonstrated^32^. However, *Xie Y. et al*.^30^ only study the apparent negative refraction corresponding to a specific high diffraction order and no more discussion about the reflection behaviors of such gradient acoustic metasurface. Therefore, the reflection behaviors of a reflected gradient acoustic metasurface beyond the critical angle is of great interest to be studied.

In this paper, we theoretically study the influences of the supercell periodicity on the reflection behaviors of the gradient acoustic metasurface. The apparent negative reflection occurs when the incident angle is beyond the critical angle, which has been perfectly verified by the calculated reflected acoustic field of the reflected gradient acoustic metasurface. The theoretical study shows that the generalized law of reflection still works within the region defined by the critical angle, however, when the incident angle is beyond the critical angle, the generalized law of reflection would be modified with the reciprocal lattice vector corresponding to the higher order diffraction. As for the possible higher order diffraction, it depends on the amplitude of the metasurface’s surface phase gradient and the incident angle, which can be regarded as a more general case discussed in ref. 30.

## Results

### The generalized law of reflection for full-angle incidence and arbitrary gradient metasurface

Since the gradient metasurface introduces the phase discontinuity across the scattered interface, the local graded phase modulation of the periodic supercell resets the classical Snell’s law by adding a surface phase gradient term which now is known as the generalized law of reflection and refraction. For a reflected gradient metasurface with a surface phase gradient 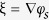, where *φ*_*s*_ is the position dependent reflected phase modulation along the gradient metasurface, the generalized law of reflection provides a complete description about the reflection behaviors of the metasurface under the plane wave illumination:


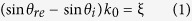


where *θ*_*i*_ is the incident angle, *θ*_*re*_ is the anomalous reflected angle and *k*_0_ is the amplitude of the free space wave vector. Since the gradient metasurface offers a linear phase modulation from 0 to 2π over a supercell period, the surface phase gradient term can be further written as 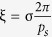, where *p*_*s*_ is the supercell period length and σ = 1 or −1, which indicates the direction of the surface phase gradient. According to formula (1), the critical angle 
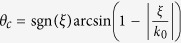
 can be solved mathematically and it defines the domain of the incident angles that satisfy the momentum matching condition along the metasurface. The gradient metasurface has a saw tooth phase modulation profile, which is exactly coincident with that of a blazed diffraction grating, and thus the anomalous reflected beam can be regarded as the +1 order diffraction as well^33^. However, the gradient metasurface is a periodic array of the inhomogeneous supercells, the non-local effect that originates from the supercell periodicity should be considered as an indispensable factor when we want to have a comprehensive understanding of the wavefront manipulation behaviors of a gradient metasurface. In this case, the generalized law of reflection can be rewritten as (exactly the same form of the formula between the incident angle and refracted angle for a transmissive gradient metasurface placed in homogeneous background medium^30^)


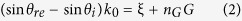


where 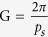 is the amplitude of the reciprocal lattice vector, and *n*_*G*_ indicates the corresponding diffraction order. According to the classical diffraction theory, *n*_*G*_ is equal to 0 as long as the incident angle is inside the region defined by the formula (1). When the incident angle is beyond the critical angle, *n*_*G*_ would take some other values to maintain the validity of formula (2) (for example, *n*_*G*_ = −3 in ref. 30), that means the anomalous reflection or the apparent negative reflection occurs.

**Figure 1:**
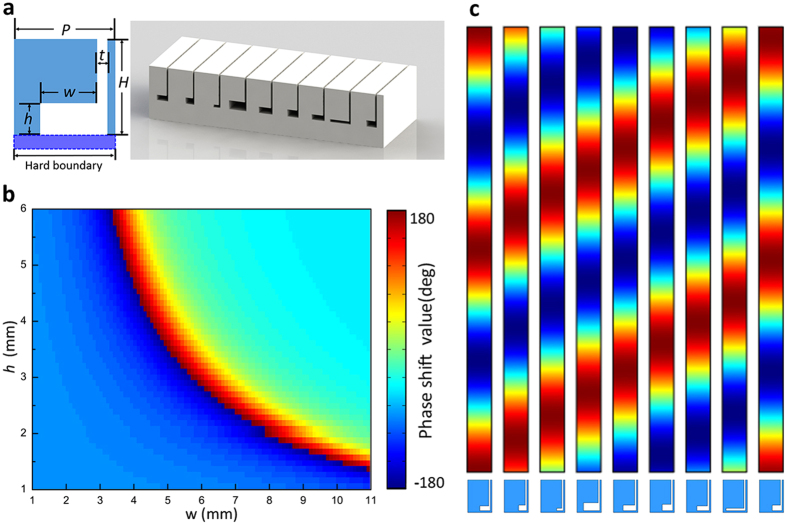
Design of the functional acoustic meta-atoms. (**a**) Schematic demonstration of the designed subunit sample and a typical supercell consists of 9 subunits. (**b**) Phase shift values map of the subunits of varying cavity width *w* and height *h*. Here, the subunit width *P* = 15 mm, neck thickness *t* = 1 mm and neck height *H* = 19 mm. (**c**) Scattered acoustic field of a typical set of subunits with a linearly varying phase modulation ranging from 0 to 2π.

For an electromagnetic metasurface composed by the gradient metallic subunits, the surface bounded wave, which is stimulated by the beam of the incident angle that is beyond the critical angle, would be wore down and transformed into the joule heat because of the intrinsic loss of the metasurface^28^,^29^. However, the acoustic energy loss along with the interaction between the incident acoustic waves and metasurface structure is determined by the background medium loss caused by medium viscosity and thermal acoustic effects. In this paper, we focus on the reflected acoustic gradient metasurface made of hard material under audible airborne (2.5 kHz) acoustic wave illumination. The significance of the influences of the viscosity and thermal acoustic effects are determined by whether the minimal geometrical parameter (for example, the width of the channel) is comparable to the acoustic boundary layer thickness or not^34^: the viscous boundary 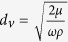 and thermal boundary 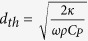, where μ is the dynamic viscosity of air, κ is the thermal conduction coefficient of air, ρ is the density of air, *C*_*p*_ is the specific heat at constant pressure, and ω is the angular frequency. In this paper (2.5 kHz, 20 °C, 1 atm.), *d*_*ν*_ = 0.044 mm, *d*_*th*_ = 0.05 mm and the minimal geometrical size is 1 mm (the thickness of the neck slit, detailed information about the meta-atoms we utilize for the full-wave simulation is presented in the following content, see Fig. 1(a)). Therefore, the intrinsic loss of the gradient acoustic metasurface can be regarded as a negligible factor when dealing with the interaction between the impinging waves and metasurface structures. Because of the low energy loss, the acoustic surface bounded waves would efficiently interact with the intrinsic Floquet-Bloch modes offered by the gradient metasurface’s periodic supercell array, and reradiate into the free space which finally leads to the apparent negative reflection governed by formula (2).

### Design of the reflected gradient metasurface for airborne sound

Following the strategy of engineering the impedance profile of the acoustic metasurface, we utilize the Helmholtz resonator with a L shape cavity as the building block to construct the desired reflected acoustic gradient metasurface. Figure 1(a) demonstrates one meta-atom sample and a typical gradient supercell composed by nine subunits which corresponds to a linear phase shift covering the full 2π range. The L shape Helmholtz resonator can be regarded as a combination of the following two parts: a vertical thin neck channel of thickness *t* and height *H*, a resonant cavity of height *h* and width *w*. In this paper, the metasurface is designed to operate under 2.5 kHz airborne acoustic wave illumination and the subunit is made of nylon which is stiff enough to treat the interaction boundary as the hard boundary. Then we calculate the scattered acoustic field of the periodic array of a single subunit, which is excited by a normally incident plane acoustic wave, and vary the height as well as the width of the bottom cavity to obtain a complete phase modulation map of the L shape Helmholtz resonator (see Fig. 1(b)). Figure 1(c) illustrates the scattered pressure field of nine subunits with the linearly varying phase modulation ranging from 0 to 2π, and the scattered field is normalized by the amplitude of the background field.

### Simulation verification of the apparent negative reflection

**Figure 2:**
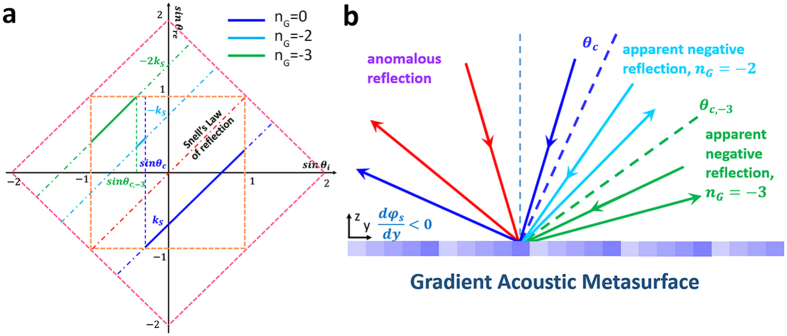
Schematic demonstration of the general case of the apparent negative reflection with the gradient acoustic metasurface. (**a**) Relation between sin *θ*_*i*_ and sin *θ*_*re*_. The blue, cyan and green solid line correspond to *n*_*G*_ takes the value of 0, −2 and −3 respectively, which represent the intrinsic anomalous reflection state 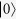 and the another two allowed apparent negative reflection states 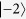 and 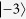 correspondingly. The red dotted line represents the specular reflection state 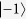, which is forbidden here. The orange dotted square circles the domain of all possible reflection states and the pink dotted square defines the all allowed phase gradient shift that maintains the anomalous wavefront manipulation characteristic of the gradient metasurface, when the amplitude of the surface phase gradient is greater than 2*k*_0_, the structured gradient metasurface works as a uniform flat impedance ground. (**b**) Schematic illustration of the anomalous reflection (red and blue solid beam), the apparent negative reflection taking place beyond the critical angle 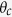 when *n*_*G*_ = −2(cyan solid beam) and *n*_*G*_ = −3(green solid beam).

To verify the validity of our theoretical prediction about the apparent negative reflection, the high order diffraction that occurs when the incident angle is beyond the critical angle, we investigate the reflection behaviors of four reflected acoustic gradient metasurfaces with different supercell periods. In this work, we assume that the reciprocal lattice vector G heads to the same direction with the surface phase gradient ξ and *n*_*G*_ is a nonpositive integer. Therefore, 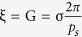, formula (2) can be rewritten as


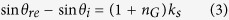


where 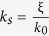 denotes the reduced surface gradient. Based on formula (3), the wave reflection behaviors of the gradient metasurface can be straightforward demonstrated by depicting the relation between sin *θ*_*i*_ and sin *θ*_*re*_. Figure 2(a) illustrates a general case of the apparent negative reflection of the reflected gradient acoustic metasurface. The orange dash line circles the domain of all possible (sin *θ*_*i*_, sin *θ*_*re*_) solutions of the allowed *k*_*s*_, and the generalized law of reflection that is amended by the reciprocal lattice vector of the supercell period can be visually demonstrated as shifting the Snell’s law of reflection within the region encompassed by the pink dashed square as well. The blue solid line shown in Fig. 2(a) indicates the anomalous reflection governed by the generalized law of reflection when the incident angle is within the critical angle. Things turn out to be interesting when the incident angle is beyond the critical angle *θ*_*c*_, the surface bounded waves supported by the linear local phase modulation of the subunits would interact with the Floquet-Bloch modes offered by the supercell lattices and reradiate into the free space as the high order diffractions. Under this case, the relation between the incident angle and reflected angle can be described by formula (3), and there could be several possible *n*_*G*_ values as long as the momentum matching condition over the metasurface is satisfied. It is apparent that the reduced surface phase gradient *k*_*s*_ of smaller absolute value will have more possible *n*_*G*_ values, and thus, to simplify our discussion, we firstly study the case that 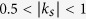. In this situation, all possible *n*_*G*_ values can be solved by maintaining the validity of formula(3) and consequently *n*_*G*_ could take the value of 0, −1, −2 and −3. Since the lateral wave vector component of the impinging wave 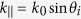 is incident angle dependent, the choice of *n*_*G*_ value would be incident angle dependent as well. When the incident angle gradually varies from 90° to −90°, the value of *n*_*G*_ can be determined by the ‘jump-up’ rule in our paper. Here we firstly treat the reflection behaviors of the gradient acoustic metasurface as several reflection states 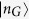, i.e., state 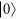 corresponds to the anomalous reflection governed by the generalized law of reflection, state 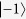 corresponds to the specular reflection which indicates that the gradient metasurface has no anomalous manipulation effects on the impinging waves, state 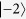 and 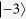 correspond to the allowed apparent negative reflections involving the higher order diffractions. When the incident angle *θ*_*i*_ varies beyond the critical angle *θ*_*c*_, reflection state 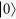 would be switched into reflection state 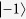, 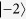 and 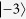. This switching procedure can be qualitative understood as the energy of the acoustic surface bounded wave redistributes into higher order diffractions via the scattering effects introduced by the supercell lattices, and within a narrow region around the critical angle, several reflection states could coexist but differ in intensity (detailed discussion is presented in Discussion part). However, the transition between different reflection states when the incident angle varies monotonously around the critical angle is relative drastic, and that is why we describe the switch between the reflection states as ‘jump-up’. It should be noted that when the incident angle domain of two allowed reflection states overlap (exclude the reflection state 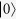), the allowed reflection state of larger absolute *n*_*G*_ value would be superior to that of a smaller one. For example, Fig. 2(a) illustrates the situation that when the incident angle varies beyond the critical angle, the anomalous reflection state 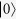 jumps to the apparent negative reflection state 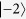 but not the mirror reflection state 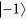, because the incident angle domain of mirror reflection state 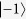 overlaps with the state 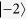. Similarly, when the incident angle is beyond the shift critical angle 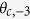 (the mathematically allowed critical angle for the corresponding high order diffraction), the apparent negative reflection state 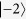 would jump to the apparent negative reflection state 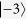 for the same reason. Figure 2(b) schematically depicts the anomalous reflection behaviors of the gradient acoustic metasurface that supports 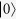, 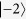 and 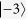 reflection state simultaneously.

**Figure 3:**
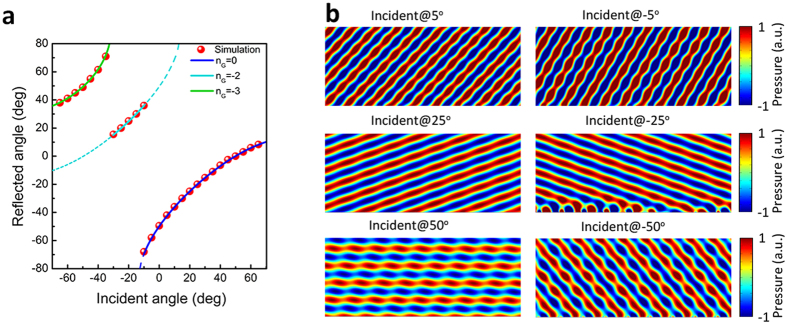
The apparent negative reflection behavior of the gradient acoustic metasurface when ξ = −0.762 *k*_0_. (**a**) The relation between the incident angle and reflected angle when *k*_*s*_ = −0.762. The red dots are data obtained from the full-wave simulation. The blue solid line indicates the anomalous reflection state 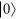, the cyan and green solid lines represent the allowed apparent negative reflection states 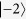 and 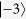 respectively. (**b**) Calculated scattered acoustic field under the plane acoustic wave illumination of different incident angles.

Figure 3 demonstrates the apparent negative reflection behaviors of the gradient acoustic metasurface with the reduced surface phase gradient *k*_*s*_ = −0.762. The critical angle *θ*_*c*_ is solved to be −13.8° and the shift critical angle 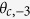 can be calculated by 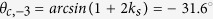. When −13.8° < *θ*_*i*_ < 90°, the reflected angle can be predicted by 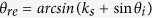, just as the blue solid line presented in Fig. 3(a). When −31.6° < *θ*_*i*_ < −13.8°, the Floquet-Bloch mode with 2G propagation constant amplitude would strongly scatter the surface bounded waves and forms the free space propagating waves, the reflected angle can be solved by 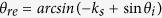. Similarly, when −90° < *θ*_*i*_ < −31.6°, the Floquet-Bloch mode with 3G propagation constant amplitude scatters the surface bounded waves intensively and the reflected angle 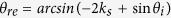. Obviously, the reflected angles that obtained from the calculated far field distribution agree well with the values calculated by formula (3). Figure 3(a) shows the apparent negative reflections corresponding to *n*_*G*_ = −2 and *n*_*G*_ = −3, Fig. 3(b) shows the calculated reflected acoustic field corresponding to different incident angles, when the plane acoustic wave is incident at 5°, −5°, 25°, −25°, 50° and −50° respectively, the beam would reflect at −42°, −58°, −20°, 20°, 0°, and 49° correspondingly.

**Figure 4:**
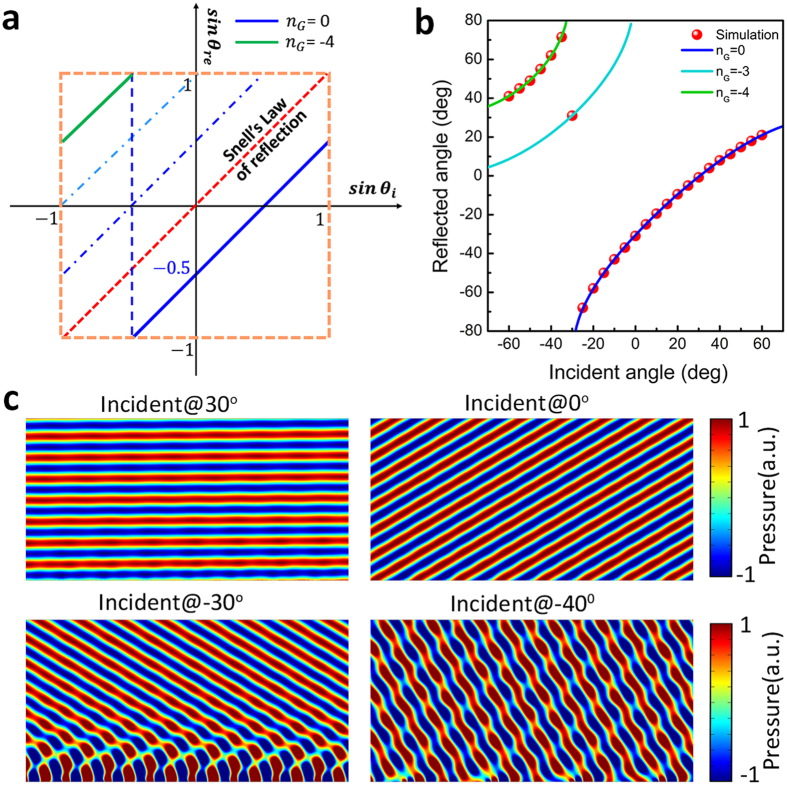
The apparent negative reflection behavior of the gradient acoustic metasurface when ξ = −0.508 *k*_0_. (**a**) Relation between sin *θ*_*i*_ and sin *θ*_*re*_ when *k*_*s*_ = −0.508. The blue solid line indicates the anomalous reflection state 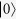, the green solid line represents the allowed apparent negative reflection 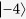. The red dotted line represents the specular reflection 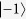, the blue and cyan dash-dot line correspond to the forbidden apparent reflection state 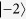 and 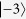. (**b**) The relation between the incident angle and reflected angle when *k*_*s*_ = −0.508. (**c**) Calculated reflected acoustic field of the gradient acoustic metasurface when *k*_*s*_ = −0.508. When the acoustic plane wave is incident at 30°, 0°, −30° and −40°, the beam would reflect at −0.8°, −31°, 31° and 62° correspondingly.

Considering the situation that 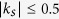, even larger absolute value of *n*_*G*_ is allowed. For example, when *k*_*s*_ =−0.5, there are five possible reflection states: 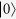, 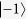, 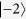, 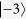, 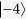. Based on the ‘jump-up’ rule, the anomalous reflection state would jump to the apparent negative reflection state 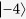 when the incident angle is beyond the critical angle (see Fig. 4(a)), because the shift critical angle 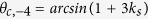 is equal to the critical angle 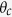. Figure 4(b) shows the well agreement between the simulation results and theoretical calculated values. And it can be seen from the picture that the apparent negative reflection state 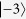 still exists, the possible reason is that the normalized surface phase gradient value of the gradient acoustic metasurface is chosen as *k*_*s*_ = −0.508, which slightly diverts from −0.5, for the full-wave simulation. However, such minor difference would shift 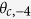 form −30° to −31.6° and *θ*_*c*_ from −30° to −29.5°, and thus the apparent negative reflection state 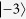 could survive in a narrow domain. Figure 4(c) shows the calculated acoustic field when the plane acoustic waves illuminate from different directions.

**Figure 5:**
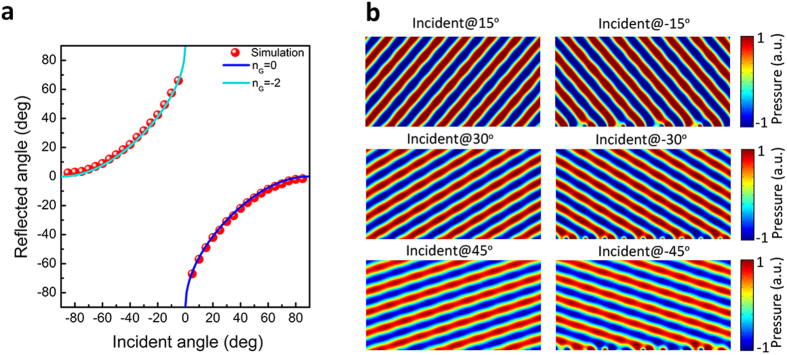
The apparent negative reflection behavior of the gradient acoustic metasurface when ξ = −1.016 *k*_0_. (**a**) The relation between the incident angle and reflected angle when *k*_*s*_ = −1.016. (**b**) Calculated scattered acoustic field under the plane acoustic wave illumination of different incident angle values when *k*_*s*_ = −1.016. The acoustic plane wave is incident at 15°, −15°, 30°, −30°, 45° and −45°, the beam would reflect at −49°, 49°, −31°, 31°, −18° and 18° correspondingly.

When 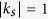, only three possible reflection states are allowed: 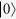, 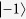 and 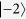. Since the shift critical angle is 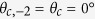, thus the anomalous reflection state 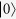 would directly jump to the apparent negative reflection state 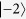 when the incident angle is beyond the critical angle, or equally saying that the surface bounded waves would strongly interact with the Floquet-Bloch mode of 2 G propagation constant amplitude, and form the apparent negative reflection beam. The center symmetrical reflected angle distribution shown in Fig. 5(a) suggests that the full-angle negative reflection can be realized by such gradient acoustic metasurface, of which the amplitude of the surface phase gradient is equal to the free space wave vector *k*_0_. When 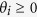, the reflected angle can be solved by 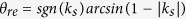 and when 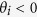, the reflected angle is 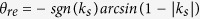. Figure 5(b) is the calculated reflected acoustic field under the illumination of the acoustic plane waves with different incident angles.

**Figure 6:**
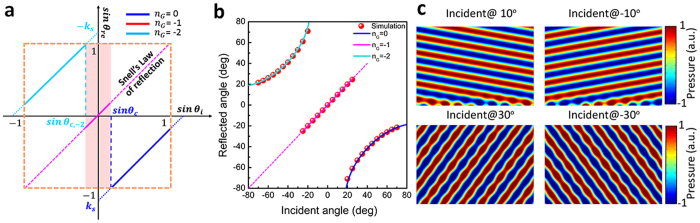
The apparent negative reflection behavior of the gradient acoustic metasurface when ξ = −1.307 *k*_0_. (**a**) The relation between sin *θ*_*i*_ and sin *θ*_*re*_ when *k*_*s*_ = −1.307. The blue solid line represents the anomalous reflection 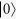, the pink solid line represents specular reflection 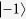 and the cyan solid line represents the apparent negative reflection 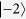. (**b**) The relation between the incident angle and reflected angle when *k*_*s*_ = −1.307. (**c**) Calculated scattered acoustic field under the plane acoustic wave illumination of different incident angle values when *k*_*s*_ = −1.307. The acoustic plane wave is incident at 10°, −10°, 30° and −30°, and the beam reflects at 10°, −10°, −53° and 53° correspondingly.

When 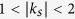, there are three possible reflection states: 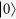, 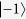 and 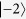. Figure 6 illustrates the reflection behaviors of the gradient acoustic metasurface with *k*_*s*_ = −1.307. The shift critical angle is 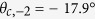 while the critical angle is 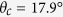. And the anomalous reflection state 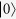 will firstly jump to the mirror reflection state 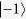, and then from state 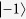 jumps to the apparent negative reflection state 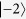 (see Fig. 6(a)). Such reflection manipulation behavior is the typical wide-angle negative reflection phenomena^35^. Figure 6(b) is the reflected angle distribution when incident angle varies from 80° to −80°. Figure 6(c) is the calculated reflected acoustic field distribution when the plane acoustic waves are incident at different angles. When 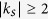, the metasurface has no manipulation effect on the incident beam and only mirror reflection occurs.

## Discussion

**Figure 7:**
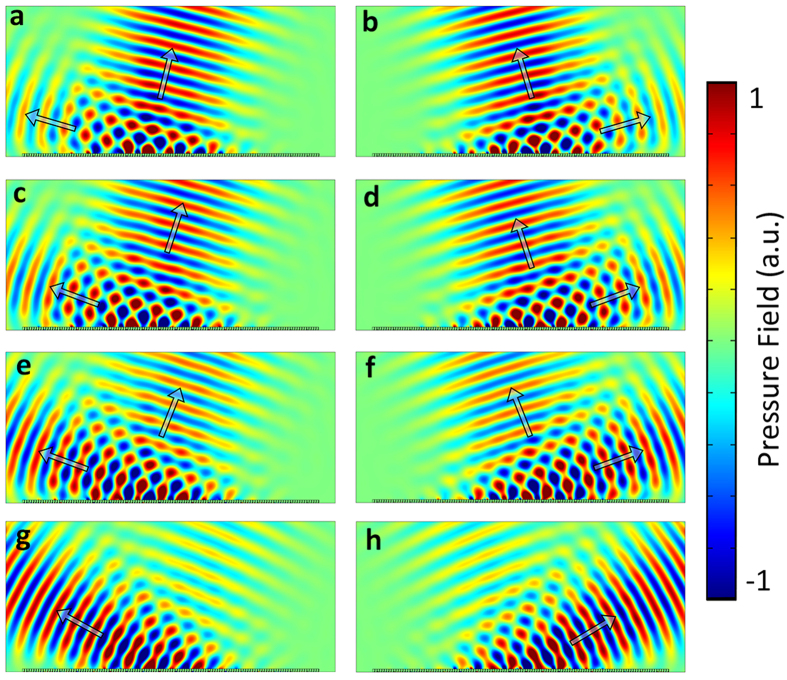
The calculated scattered acoustic field when the acoustic Gaussian beam impinges onto a finite gradient metasurface composed by 18 supercells, each supercell consists of 7 subunits which corresponds to a −1.307 *k*_0_ surface phase gradient. (**a**)~(**h**) are the scattered field when the incident angle is 15°, −15°, 17°, −17°, 20°, −20°, 25° and −25° respectively.

The ‘jump’ between the different reflection states is the redistribution of the reflected acoustic energy of different diffraction orders, which originates from the interaction between the surface bounded waves and the effect of the non-local supercell period. It can be seen from the calculated reflected acoustic field corresponding to the apparent negative reflection, the total scattered field is the superposition of a surface bounded waves and the free space propagation wave (see Fig. 5(b)). When we vary the incident angles around the critical angle or the shift critical angle, we can observe a gradual evolution of the redistributed acoustic energy between different reflection states. For example, Fig. 6(b) indicates that when the incident angle locates around the critical angle 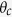 or the shift critical angle 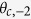, one incident angle could correspond to two reflected angles simultaneously, because the two reflection states have the comparable field intensity on this occasion. Figure 7 shows a more straightforward illustration of the energy redistribution between different reflection states when an acoustic Gaussian beam of gradually varying incident angles impinges upon the gradient metasurface of the finite length (the energy redistribution between the anomalous reflection state 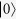 and the specular reflection state 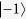 around the critical angle 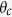, see Fig. 7(a), (c), (e) and (g); the energy redistribution between the specular reflection state 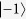 and apparent negative reflection 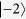 around the shift critical angle 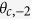, see Fig. 7(b), (d), (f) and (h)).

It should be noted that such apparent negative reflection and the anomalous reflection phenomenon of the gradient acoustic metasurface could distort the scattered field of the object encompassed by it and thus the gradient acoustic metasurface has the potential to be applied as the illusion devices. Furthermore, the full-angle negative reflection phenomena illustrated in Fig. 5 indicates the unique trait of an acoustic insulator: when two acoustic beams are incident from opposite direction, the reflected beams locate at the same quadrant with the incident beam, therefore, two reflected beams would not encounter.

## Conclusion

In summary, we demonstrate the apparent negative reflection phenomenon that takes place when the incident angle is beyond the critical angle, which should be forbidden according to the generalized law of reflection, can be realized with the reflected gradient acoustic metasurface. The underlying mechanism of such anomalous phenomenon can be understood by treating the apparent negative reflection as the high order diffraction which is produced via the interaction between the surface bounded waves and the supercell periodicity. Integrating the reciprocal lattice vector term, which represents the influences of the supercell periodicity, to the generalized law of reflection offers perfect prediction about the reflected angle values of the apparent negative reflection. Full-wave simulation of the scattered acoustic fields of the gradient acoustic metasurface illustrates versatile apparent negative reflection phenomenon perfectly, and the nontrivial full-angle negative reflection, which combines the anomalous reflection and the apparent negative reflection behaviors of the gradient acoustic metasurface, offers a simple way for isolating the acoustic waves incident from opposite directions. This study may provide more freedom for designing the acoustic signal modulation devices and the potential applications in acoustic beam steering.

## Methods

In this paper, we take advantage of the Finite Element Method (FEM) based on the commercial software COMSOL Multiphysics to conduct the full-wave simulations. The acoustic pressure model is used, the background medium is set to be air and the corresponding acoustic speed is chosen as 343 m/s. The gradient metasurface is studied as the infinite period by applying the periodic boundary condition on the right and left sides of the simulation region. The reflected angle is obtained according to the far-field acoustic pressure distribution. As for the influences of the viscosity and thermal acoustic effects on the reflection behaviors of the metasurface, we utilize two different methods to testify the robustness of the apparent negative reflection by introduce an imaginary part to the acoustic refractive index of the air 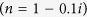 or directly apply the thermal acoustic model within the metasurface structures to conduct the full-wave simulation. The calculated reflected acoustic fields obtained by the above methods are almost same with the results that calculated without considering the dissipative loss.

## Additional Information

**How to cite this article**: Liu, B. *et al*. Apparent Negative Reflection with the Gradient Acoustic Metasurface by Integrating Supercell Periodicity into the Generalized Law of Reflection. *Sci. Rep.* **6**, 38314; doi: 10.1038/srep38314 (2016).

**Publisher's note:** Springer Nature remains neutral with regard to jurisdictional claims in published maps and institutional affiliations.
